# Retinoic Acid Signaling in Male Reproductive Biology: From Germ Cell Regulation to Contraceptive Innovation Within a One Health Framework

**DOI:** 10.3390/ani16121831

**Published:** 2026-06-14

**Authors:** Vanmathy Kasimanickam, Ramanathan Kasimanickam

**Affiliations:** 1 Independent Researcher, 705, NW Valley View Drive, Pullman, WA 99163, USA; 2 College of Veterinary Medicine, Washington State University, Pullman, WA 99164, USA

**Keywords:** retinoic acid, canine testis, spermatogenesis, *STRA8*, *CYP26B1*, *ALDH1A2*, *RARα*, microRNA, male contraception, reproductive biology

## Abstract

Spermatogenesis is a highly coordinated biological process in which diploid spermatogonia undergo mitotic expansion, meiotic division, and terminal differentiation into haploid spermatozoa. This process is tightly regulated by intrinsic germ cell programs and extrinsic signals from Sertoli cells within the seminiferous epithelium. Among the signaling pathways governing male germ cell development, all-trans retinoic acid (RA), a bioactive metabolite of vitamin A, has emerged as a master regulator of meiotic initiation and spermatogonial differentiation in mammals. RA functions through nuclear retinoic acid receptors (RARs) and retinoid X receptors (RXRs), which regulate transcriptional networks essential for germ cell progression, including the activation of Stimulated by Retinoic Acid 8 (*STRA8*), a key determinant of meiotic entry. Intratesticular RA homeostasis is maintained by a balance between synthesis via aldehyde dehydrogenase (ALDH) enzymes and degradation by cytochrome P450 family 26 (CYP26) enzymes, ensuring precise temporal and spatial control of germ cell development. While rodent models have defined core mechanisms of RA signaling, the canine testis provides a valuable comparative and translational system due to its physiological similarity to human spermatogenesis and relevance to reproductive management. Recent studies highlight conserved RA signaling pathways in dogs, including receptor-mediated transcriptional regulation, feedback control of RA metabolism, and post-transcriptional modulation via microRNAs. Importantly, pharmacological manipulation of RA signaling can reversibly disrupt spermatogenesis, supporting its potential applications in non-hormonal male contraception. This review integrates molecular, developmental, pharmacological, and comparative evidence and presents RA signaling as a central regulatory axis of spermatogenesis with important translational applications.

## Simple Summary

Spermatogenesis is the process of making sperm in the testes. It starts with diploid spermatogonia, which multiply, then undergo meiosis, and finally develop into haploid sperm cells. This process is controlled by internal germ cell programs and by Sertoli cells that support development. A major regulator is all-trans retinoic acid (RA), a form of vitamin A. RA triggers the start of meiosis and helps spermatogonia mature by activating important genes such as Stimulated by Retinoic Acid 8 (*STRA8*). RA acts through nuclear receptors that control gene expression. Its levels in the testes are tightly regulated by enzymes that both produce and break down RA, ensuring sperm develop at the correct time and place. Research in rodents and dogs shows that RA signaling is conserved across mammals, with dogs providing a useful model for human reproduction. Disrupting RA signaling can temporarily stop sperm production, suggesting potential for non-hormonal male contraception and other reproductive medicine applications.

## 1. Introduction

Spermatogenesis is a highly coordinated and tightly regulated developmental process through which diploid spermatogonia undergo mitotic proliferation, meiotic division, and terminal differentiation to generate haploid spermatozoa capable of fertilization [1,2,3]. This complex biological program occurs within the seminiferous epithelium of the testis and relies on precise temporal and spatial interactions between developing germ cells and surrounding somatic cells, particularly Sertoli cells, which provide structural, nutritional, and endocrine support essential for germ cell survival and maturation [4,5,6]. The successful progression of spermatogenesis is fundamental to male fertility, and disturbances in the signaling pathways regulating germ cell proliferation, meiotic entry, or differentiation can lead to impaired spermatogenesis, subfertility, or complete sterility [7,8]. Consequently, understanding the molecular and cellular mechanisms governing spermatogenesis has become a major focus in reproductive biology, not only to elucidate the basis of male fertility but also to identify targets for therapeutic intervention and the development of reversible male contraceptives [9,10,11,12].

Among the numerous signaling molecules involved in testicular development and germ cell maturation, all-trans retinoic acid (RA), the biologically active metabolite of vitamin A, has emerged as a master regulator of spermatogonial differentiation and meiotic initiation in mammals [13,14,15]. RA signaling is mediated through retinoic acid receptors (*RARα*, *RARβ*, and *RARγ*) and retinoid X receptors (*RXR*s), which function as ligand-activated transcription factors that bind to retinoic acid response elements within the promoters of target genes [16,17,18]. Upon activation, these receptor complexes regulate transcriptional networks required for germ cell development, including the Stimulated by Retinoic Acid gene 8 (*STRA8*), a critical factor necessary for premeiotic DNA replication and entry into meiosis [19,20]. Studies in rodent models have demonstrated that periodic pulses of RA within the seminiferous epithelium synchronize waves of spermatogenesis by coordinating *STRA8* activation and subsequent germ cell differentiation [21]. The availability of RA within the testis is tightly controlled through a balance between synthesis by aldehyde dehydrogenase (ALDH) enzymes and catabolism by cytochrome P450 family 26 (CYP26) enzymes, ensuring that intratesticular RA concentrations remain within a physiological range that supports orderly meiotic progression while preventing premature germ cell differentiation [22].

RA’s role is well characterized in rodents, but its study in other mammals advances comparative understanding. Rodent studies have revealed key mechanisms of RA-mediated spermatogenesis. However, the canine testis is now recognized as an important comparative and translational model for reproductive research [3,23,24]. Dogs share reproductive traits with humans, such as prolonged prepubertal development, complex seminiferous epithelial organization, and breed-related variation in sexual maturation [24,25]. These features make the canine model well-suited for studying species-specific germ cell regulation and bridging gaps between rodent and human systems. Canine reproductive research also offers practical benefits. The rising global dog population raises public health, ecological, and animal welfare issues, driving a need for safe, effective, reversible non-surgical male contraception [26,27]. Understanding the molecular regulation of spermatogenesis in dogs may not only clarify fundamental biology but also help develop new fertility-control strategies.

Recent advances in canine reproductive research have significantly expanded current understanding of the molecular architecture of RA signaling in the testis by applying organotypic culture systems, pharmacological manipulation, developmental profiling, and microRNA (miRNA) analyses [15,28]. Organotypic testicular culture models preserve the functional interactions between germ cells and supporting somatic cells, thereby enabling controlled ex vivo investigation of RA-dependent signaling pathways and cellular responses [15,29]. Experimental studies have shown that exogenous RA treatment or selective inhibition of RARα alters the expression of genes involved in RA metabolism, including *ALDH1* isoforms associated with RA synthesis and *CYP26B1* responsible for RA degradation, as well as downstream meiotic regulators such as *STRA8*, Deleted in Azoospermia-Like (*DAZL*), and Doublesex and mab-3 Related Transcription Factor 1 (*DMRT1*) [22]. These findings support the existence of a tightly regulated autoregulatory network in which RA modulates both its own synthesis and catabolism while simultaneously promoting meiotic competence and germ cell differentiation. Furthermore, differential expression patterns among early and late meiotic markers indicate that RA signaling is closely associated with developmental stage and germ cell progression. Beyond transcriptional regulation, RA signaling also influences post-transcriptional mechanisms by modulating conserved miRNA clusters that regulate germ cell proliferation, differentiation, and survival [30,31]. This additional post-transcriptional regulatory layer likely contributes to the temporal synchronization of spermatogenesis and may provide novel biomarkers for evaluating testicular function or responses to pharmacological intervention. Collectively, these findings establish RA as a central morphogen integrating metabolic, transcriptional, and post-transcriptional pathways to coordinate spermatogenesis. Moreover, the evolutionary conservation of major RA signaling components across mammalian species underscores the broader translational significance of the canine model for understanding male reproductive biology, as similarities and differences can guide the development of targeted approaches for fertility regulation and the treatment of testicular dysfunction [13,21,31,32].

Although the molecular framework of retinoic acid (RA) signaling has been extensively characterized in rodent models, the canine testis offers unique translational advantages that warrant greater emphasis throughout this review. Dogs exhibit prolonged prepubertal development, breed-associated variation in reproductive maturation, and spermatogenic kinetics that more closely resemble human reproductive physiology than those of rodents. Furthermore, canine-specific studies have demonstrated developmental regulation of ALDH1A isoforms and CYP26 enzymes, differential localization of RARα, RARβ, and RARγ in germ cells and spermatozoa, and modulation of meiosis-associated genes and microRNA networks following pharmacological manipulation of RA signaling. These findings establish the canine testis not merely as a comparative model but as an important translational platform for understanding species-specific regulation of spermatogenesis and evaluating reproductive interventions relevant to both veterinary and human medicine.

Several reviews have summarized the role of retinoic acid in spermatogenesis; however, this review differs in four important ways. First, it integrates canine-specific evidence rather than relying predominantly on rodent data. Second, it combines molecular, developmental, pharmacological, and microRNA-mediated aspects of RA regulation into a unified framework. Third, it evaluates RA signaling as a target for non-hormonal male contraception. Finally, it places reproductive control within a broader One Health context that links fertility management to public health, animal welfare, and environmental sustainability. These features distinguish the present review from previous reviews focused solely on basic mechanisms of RA biology.

This integrated view, which brings together developmental, pharmacological, and epigenetic evidence from the canine model, establishes a comprehensive framework for understanding RA-regulated spermatogenesis. It also highlights the potential for targeted modulation of this pathway to control fertility or treat testicular dysfunction.

## 2. Literature Search Strategy and Study Selection

This targeted narrative review synthesizes current knowledge of retinoic acid (RA) signaling in male reproductive biology, with emphasis on spermatogenesis, canine reproductive physiology, non-hormonal contraception, and One Health. Literature searches were done from January to June 2026 using PubMed, Web of Science, Scopus, and Google Scholar. Search terms included combinations such as “retinoic acid AND spermatogenesis”, “retinoic acid signaling AND testis”, “retinoic acid receptor AND male fertility”, “STRA8 AND meiosis”, “DAZL AND spermatogenesis”, “retinoic acid AND contraception”, “non-hormonal male contraception”, “retinoic acid AND dog”, “canine testis retinoic acid”, “retinoic acid AND reproductive biology”, “retinoic acid AND One Health”, and “retinoic acid AND population control”. Additional studies were found by manually reviewing the reference lists of relevant publications.

The initial search yielded about 1200 records across all databases. After duplicates were removed and titles and abstracts were screened, about 240 publications were retained for full-text evaluation. Priority was given to peer-reviewed original research, review articles, and authoritative reports in English, especially those from the past 15 years. Seminal earlier studies were included if they provided foundational insights into RA metabolism, receptor signaling, germ cell differentiation, or contraceptive development. Publications focusing on male reproductive biology, spermatogenesis, meiosis, retinoid metabolism, fertility regulation, and translational applications were considered eligible for inclusion. Studies not relevant to reproductive biology or providing insufficient mechanistic or translational information were excluded. Ultimately, 109 references were selected and incorporated into this review based on their scientific relevance, methodological quality, and contribution to understanding the biological and translational significance of RA signaling.

## 3. RA Homeostasis in the Postnatal Canine Testis

Retinoic acid (RA) homeostasis in the testis is maintained through a tightly regulated balance between synthesis and degradation, ensuring that germ cells are exposed to appropriate ligand concentrations required for differentiation and meiotic initiation [33]. Precise temporal and spatial regulation of RA signaling is essential during postnatal testicular development, as disruption of RA availability can impair spermatogenesis and compromise fertility [34]. In the canine testis, developmental and functional studies have identified coordinated metabolic pathways that regulate RA production and catabolism, establishing a dynamic homeostatic network that supports orderly germ cell progression [22].

### 3.1. RA Synthesis: ALDH Isozymes

The synthesis of biologically active RA from retinaldehyde is catalyzed by ALDH isozymes, primarily ALDH1A1, ALDH1A2, and ALDH1A3 [35]. Developmental profiling of the canine testis has demonstrated differential expression of these enzymes across prepubertal, peripubertal, and adult stages of maturation [22]. Among there enzymes, ALDH1A2 appears to be the predominant isozyme associated with the onset of active spermatogenesis, suggesting that it serves as the principal source of RA required for spermatogonial differentiation and meiotic initiation [36,37]. Increased *ALDH1A2* expression coincides with the acquisition of meiotic competence in germ cells, indicating that RA synthesis is developmentally synchronized with germ cell maturation [14,37].

Functional studies using organotypic testicular cultures further revealed that exogenous RA suppresses *ALDH1* expression in a dose-dependent manner [15,22,38]. This negative feedback mechanism suggests that RA autoregulates its own biosynthesis to prevent excessive accumulation and maintain physiological signaling thresholds necessary for coordinated spermatogenic progression [22,38]. Therefore, ALDH isozymes not only generate RA required for meiotic entry but also participate in feedback-regulated control of RA availability within the seminiferous epithelium [15,22,39].

### 3.2. RA Catabolism: CYP26 Enzymes

RA degradation is mediated primarily by cytochrome P450 family 26 (CYP26) enzymes, which convert RA into inactive metabolites [22,39,40]. In the canine testis, CYP26B1 has been identified as the major RA-catabolizing enzyme throughout postnatal development [22]. Elevated *CYP26B1* expression during periods of active spermatogonial differentiation indicates that regulated RA degradation is critical for restricting RA exposure spatially and temporally within the seminiferous epithelium [31,41].

*CYP26B1* expression itself is regulated by RA signaling. Exogenous RA stimulates CYP26B1 transcription, whereas inhibition of RARα suppresses its expression [22,31,41,42]. This feedback loop enables RA to promote its own degradation, thereby preventing excessive activation of downstream meiotic regulators such as *STRA8*. The coordinated interaction between ALDH-mediated synthesis and CYP26B1-dependent catabolism functions as a metabolic rheostat that maintains intratesticular RA concentrations within an optimal physiological range necessary for normal spermatogenesis [22,31,41,42,43].

## 4. RA Receptor Signaling and Downstream Effectors in the Canine Testis

Retinoic acid (RA) regulates spermatogenesis primarily through nuclear retinoic acid receptors (RARα, RARβ, and RARγ) and their heterodimeric partners, the retinoid X receptors (RXRs) [15,44]. Following ligand binding, RAR–RXR complexes interact with retinoic acid response elements (RAREs) in target gene promoters, thereby activating transcriptional programs required for germ cell differentiation and meiotic initiation [45]. The expression and localization of these receptors within the seminiferous epithelium are tightly regulated, ensuring that RA signaling occurs in specific germ cell populations and developmental stages essential for normal spermatogenesis [46].

Immunolocalization studies in canine testis and spermatozoa have demonstrated differential distribution of RAR subtypes, indicating distinct functional roles during germ cell development [47]. RARα is predominantly localized in spermatogonia and early spermatocytes, supporting its central role in mediating RA-dependent meiotic initiation. In contrast, RARγ is enriched in later-stage spermatocytes and spermatids, suggesting involvement in post-meiotic differentiation and chromatin remodeling. RARβ expression is comparatively lower but remains detectable, implying potential context-dependent or compensatory functions. Comparative analyses with rodent and bovine models indicate that the overall organization of RA receptor signaling is evolutionarily conserved, although species-specific differences in receptor localization and developmental timing may influence the dynamics of canine spermatogenesis [15,47].

Activation of RARs induces the expression of downstream effectors essential for meiotic progression. Among these, *STRA8* is a key RA-responsive regulator required for premeiotic DNA replication and chromosomal synapsis. In canine organotypic testicular cultures, exogenous RA and pharmacological modulation of *CYP26B1*-mediated RA metabolism significantly increase *STRA8* expression, confirming the sensitivity of this pathway to RA availability [48]. Additional RA-responsive factors include *DAZL*, an RNA-binding protein necessary for germ cell differentiation and *STRA8* induction, and *DMRT1*, which integrates RA signaling to regulate the balance between spermatogonial proliferation and differentiation [49,50,51]. Together, these molecules form an interconnected regulatory network linking receptor activation to meiotic competence and germ cell maturation.

Beyond transcriptional regulation, RA signaling also modulates post-transcriptional pathways through regulation of microRNA (miRNA) expression. Conserved miRNA families, including miR-200, let-7, and miR-34, are upregulated in response to RA and contribute to fine-tuning genes associated with meiotic progression, differentiation, and germ cell survival [31]. Conversely, suppression of specific miRNAs relieves repression of RA-responsive transcripts, further promoting differentiation. Pharmacological inhibition of RARα reduces *STRA8*, *DAZL*, and *DMRT1* expression in organotypic cultures, demonstrating that intact receptor signaling is essential for normal germ cell development [22,31,52,53]. Collectively, these findings establish RA receptor signaling as a multi-layered regulatory system integrating transcriptional and post-transcriptional mechanisms to coordinate spermatogenesis in the canine testis while also highlighting potential targets for fertility regulation and treatment of testicular dysfunction.

## 5. Pharmacological Modulation of RA Signaling and Translational Implications

The tightly regulated retinoic acid (RA) signaling network in the canine testis presents multiple targets for pharmacological manipulation, providing opportunities to modulate spermatogenesis with temporal and mechanistic specificity. Experimental studies have investigated strategies aimed at altering ligand availability, inhibiting RA catabolism, or blocking receptor activity, each producing distinct effects on germ cell differentiation and meiotic progression [22,31,41]. These approaches have important implications for reproductive biology, reversible male contraception, and treatment of testicular dysfunction.

### 5.1. Exogenous RA Supplementation

Administration of all-trans RA in canine organotypic testicular cultures increases expression of key meiotic regulators, including *STRA8*, *DAZL*, and *DMRT1*, confirming the responsiveness of the canine testis to enhanced RA signaling [22,31,52,53]. Exogenous RA also induces expression of *CYP26B1* and Cellular Retinoic Acid-Binding Protein II (CRABPII), indicating activation of intrinsic feedback pathways that regulate ligand availability [22,31]. However, the effectiveness of direct RA supplementation is limited by rapid metabolic degradation through CYP26-mediated catabolism, which restricts both the magnitude and duration of signaling. These findings emphasize the importance of controlled dosing and exposure timing when considering RA supplementation as a strategy for manipulating spermatogenesis.

### 5.2. RA Catabolic Inhibition

An alternative approach involves inhibition of *CYP26B1*, the primary RA-catabolizing enzyme in the canine testis. Pharmacological inhibition using the selective CYP26 inhibitor R115866 produces marked upregulation of *STRA8* and other meiotic competence factors, often exceeding the effects observed following direct RA supplementation [48,54,55]. By preventing RA degradation, *CYP26B1* inhibition sustains endogenous RA concentrations and prolongs signaling activity within the seminiferous epithelium. This strategy enhances coordinated germ cell differentiation while avoiding the rapid clearance associated with exogenous ligand administration, suggesting that modulation of RA catabolism may represent a more efficient and physiologically relevant method for regulating spermatogenesis.

### 5.3. RAR Antagonism

Selective inhibition of RARα using antagonists such as Ro 41-5253 suppresses RA-dependent signaling by reducing receptor-mediated transcriptional activation [15]. In canine organotypic cultures, RARα antagonism decreases expression of *STRA8*, *DAZL*, and *DMRT1*, effectively blocking meiotic initiation and germ cell differentiation [22,31,52,53]. These findings identify RARα as a critical regulatory node in RA-mediated spermatogenesis and support its potential as a target for reversible male contraception. Nevertheless, prolonged receptor inhibition may interfere with broader RA-dependent processes, including Sertoli cell support functions and post-meiotic germ cell maturation, highlighting the need for careful temporal and dosage control.

### 5.4. Translational Implications and Future Perspectives

The ability to pharmacologically manipulate RA signaling has significant translational relevance, particularly for the development of non-surgical male contraceptive strategies in dogs, where population control remains a major public health and animal welfare concern [22,31,41,43,55,56]. Compared with hormonal castration, RA-targeted approaches may provide reversible and mechanistically specific suppression of spermatogenesis with fewer systemic effects. In addition, modulation of RA pathways may offer therapeutic potential for treating impaired spermatogenesis, germ cell maturation defects, and idiopathic infertility [57]. Because major components of RA signaling are conserved between dogs and humans, insights from canine studies may also contribute to the development of human reproductive therapies and contraceptive technologies.

## 6. Retinoic Acid Signaling in the Canine Testis: Contraceptive Opportunities

Retinoic acid (RA) signaling is a central regulator of spermatogenesis and represents a promising target for non-hormonal male contraception in the domestic dog. In the canine testis, RA is synthesized from dietary vitamin A through sequential oxidation reactions mediated by retinol dehydrogenases and ALDH1A enzymes within Sertoli cells and germ cells [13,22]. Pulsatile RA production regulates the differentiation of undifferentiated spermatogonia and activates retinoic acid receptors (RARα, RARβ, and RARγ), which form heterodimers with retinoid X receptors (RXRs) to induce transcription of genes essential for meiotic initiation, particularly *STRA8* [15,55]. Transcriptomic and immunohistochemical studies in the canine seminiferous epithelium demonstrate conserved expression of RARα in Sertoli cells and differentiating germ cells, supporting a model in which cyclical RA signaling coordinates stage-specific progression through the seminiferous epithelial cycle [58,59].

Experimental disruption of RA synthesis, CYP26-mediated catabolism, or receptor signaling in rodent models induces spermatogenic arrest and reversible infertility, providing strong biological support for translational application in dogs [60]. Unlike steroid-based contraceptive approaches, RA-targeted strategies act downstream of the hypothalamic–pituitary–gonadal axis and therefore preserve circulating testosterone concentrations, libido, and secondary sexual characteristics [15,61]. These characteristics are particularly advantageous for companion and working dogs where maintenance of normal behavior is desirable.

Small-molecule RAR antagonists and inhibitors of retinaldehyde dehydrogenases have demonstrated reversible suppression of spermatogenesis in preclinical models, with fertility restored following withdrawal of treatment [15,61,62,63]. In addition, inhibition of CYP26-mediated RA degradation has emerged as a potential strategy for disrupting meiotic progression through sustained alterations in endogenous RA levels. Because canine spermatogenesis exhibits species-specific kinetics and seminiferous cycle dynamics distinct from rodents and humans, pharmacodynamic studies tailored to canine physiology are necessary to optimize dosing and recovery intervals [6,64].

Targeted delivery systems, including long-acting injectable formulations and testis-targeted prodrugs, may further improve safety by minimizing systemic retinoid-associated adverse effects such as mucocutaneous toxicity and teratogenicity [65,66,67]. Collectively, current evidence identifies RA signaling as a mechanistically precise and translationally relevant target for the development of reversible, non-hormonal contraceptive strategies in dogs.

### Challenges and Considerations for RA-Based Contraceptive Development

The contraceptive potential of RA-targeted approaches should be considered within the broader context of male fertility control strategies. Although inhibition of RA signaling can reversibly suppress spermatogenesis in experimental models, practical implementation faces several challenges. Systemic retinoid exposure may result in adverse effects including mucocutaneous toxicity, hepatotoxicity, and teratogenicity. In addition, the duration of infertility, consistency of recovery following treatment withdrawal, and variability among breeds remain unknown. Therefore, future development of RA-based contraceptives will require targeted delivery systems, improved tissue specificity, and rigorous safety evaluations before field application can be considered.

**Figure 1:**
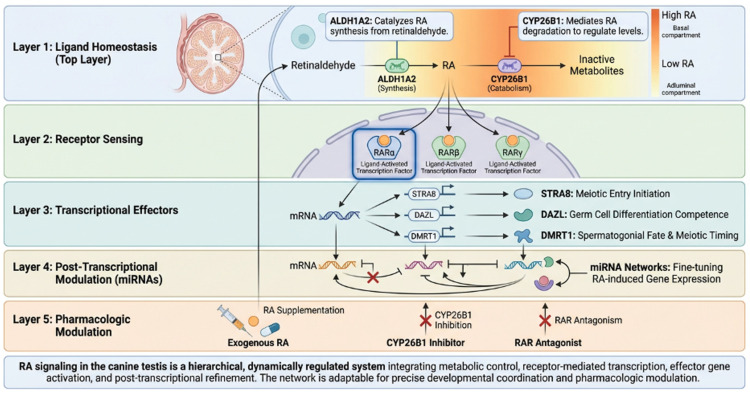
Multi-layered retinoic acid (RA) signaling network in canine testis.

Figure 1 illustrates a multi-layered retinoic acid (RA) signaling network in the canine testis, including ligand homeostasis via *ALDH1A2* and *CYP26B1*, spatial RA gradient dynamics, nuclear RA receptor (RARα, RARβ, RARγ) sensing, downstream activation of *STRA8*, *DAZL*, and *DMRT1*, post-transcriptional modulation by miRNA clusters, and targeted pharmacologic interventions affecting different network nodes. Together, these layers define RA signaling in the canine testis as a multi-tiered regulatory system integrating metabolic control, receptor-mediated transcription, effector gene activation, and post-transcriptional refinement. Rather than functioning as a linear pathway, the RA network operates as a dynamic, adaptable regulatory architecture capable of precise developmental coordination and pharmacologic modulation.

## 7. Comparative Insights and Translational Relevance

Retinoic acid (RA) signaling is a highly conserved regulator of spermatogenesis across mammals, coordinating meiotic initiation, germ cell differentiation, and maintenance of testicular homeostasis [68]. Comparative analyses among rodent, bovine, canine, and human systems have revealed a common molecular framework centered on RA synthesis, receptor-mediated signaling, and downstream transcriptional regulation, while also highlighting species-specific adaptations in spermatogenic timing and testicular organization [69,70,71,72]. These comparative studies provide important insight into reproductive biology and strengthen the translational relevance of canine models for fertility research.

### 7.1. Rodent Models: Foundational Mechanistic Insights

Rodent models have provided the foundation for understanding the molecular mechanisms of RA-mediated spermatogenesis. In mice, RA synthesized predominantly by ALDH1A2 induces expression of *STRA8* in premeiotic spermatogonia, thereby triggering meiotic initiation in a stage-dependent manner [73]. CYP26B1-mediated RA degradation restricts inappropriate meiotic entry during embryonic and postnatal development, establishing tightly coordinated spermatogenic waves [74]. Distinct expression patterns of *RARα*, *RARβ*, and *RARγ* within spermatogonia and spermatocytes further demonstrate specialized receptor functions during germ cell differentiation [60]. Rodent studies have also identified key downstream effectors, including *DAZL* and *DMRT1*, as well as RA-responsive microRNAs that fine-tune meiotic progression and germ cell maturation. Together, these findings established the canonical RA signaling pathway that serves as the basis for comparative investigations in other mammals.

### 7.2. Canine and Bovine Spermatogenesis: Conservation and Species-Specific Adaptations

Canine spermatogenesis exhibits substantial conservation of RA signaling mechanisms while also displaying distinctive developmental features. Organotypic culture and developmental studies in the canine testis demonstrate that RA regulates meiotic initiation through *STRA8* activation, with ALDH1A2-mediated synthesis and CYP26B1-dependent catabolism functioning as a tightly regulated metabolic rheostat [22,31]. Immunolocalization studies further reveal differential distribution of RARα, RARβ, and RARγ in germ cells and spermatozoa, suggesting both transcriptional and potential non-genomic roles during spermatogenesis and sperm maturation [47]. Compared with rodents, spermatogenesis in dogs is less synchronized and characterized by species-specific timing of meiotic marker expression [75]. Nevertheless, the overall RA signaling architecture remains highly conserved.

Similar patterns are observed in bovine testes, where *RARα* and *RARγ* are prominently expressed in germ cells and sperm, while *RARβ* expression remains comparatively lower [47]. *STRA8* activation and CYP26-mediated RA catabolism also parallel pathways described in canine and rodent models [48,76]. However, differences in spermatogenic kinetics and reproductive physiology suggest that local tissue organization and environmental influences may modulate RA signaling dynamics in domestic mammals [13].

### 7.3. Human Relevance and Translational Implications

The strong conservation of RA signaling components across mammals underscores the translational significance of canine studies for human reproductive biology. Human testes express *ALDH1A2*, *CYP26B1*, *RAR*s, and *STRA8* in coordinated developmental patterns like those observed in dogs and rodents [22,37,77]. Dysregulation of RA signaling has been associated with defective spermatogenesis, impaired meiotic progression, and idiopathic infertility in men [78]. Consequently, the canine model represents a valuable intermediate system bridging mechanistic rodent studies and human reproductive physiology.

Comparative studies also support the broader applicability of pharmacological approaches targeting RA signaling. Strategies such as RA supplementation, CYP26 inhibition, and RAR antagonism may provide reversible and non-hormonal approaches for fertility regulation across mammalian species [79,80]. In addition, modulation of RA-responsive transcriptional and microRNA-mediated networks may offer novel therapeutic opportunities for treating testicular dysfunction and improving assisted reproductive technologies. Collectively, these findings establish RA signaling as a conserved and translationally relevant pathway with broad implications for reproductive medicine and contraceptive development.

**Figure 2:**
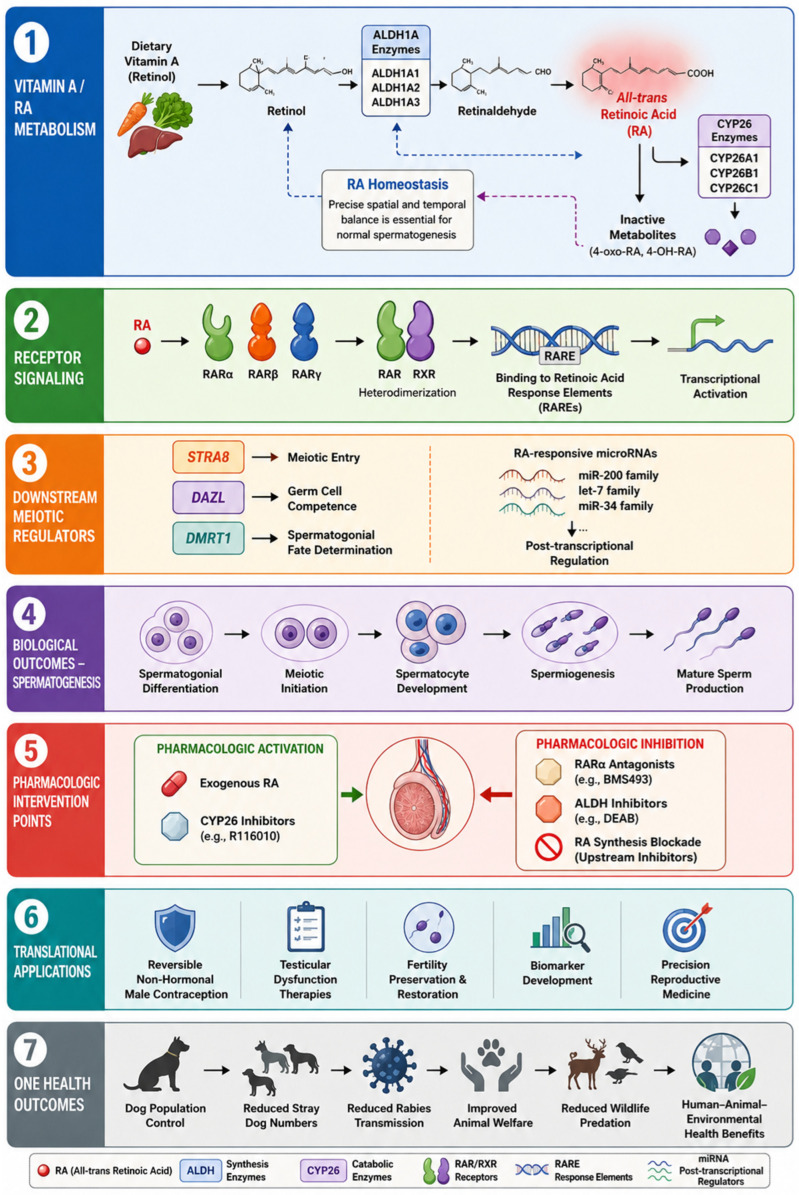
Integrated retinoic acid (RA) signaling pathway regulating spermatogenesis and its translational applications within a One Health framework. Dietary vitamin A is metabolized to all-trans retinoic acid (RA) through ALDH1A enzymes and degraded by CYP26 enzymes to maintain intratesticular RA homeostasis. RA binds retinoic acid receptors (RARα, RARβ, and RARγ) and retinoid X receptors (RXRs), activating transcriptional programs through retinoic acid response elements (RAREs). Downstream effectors, including STRA8, DAZL, and DMRT1, coordinate spermatogonial differentiation, meiotic initiation, and germ-cell maturation, while RA-responsive microRNAs provide post-transcriptional regulation. Pharmacological modulation of RA signaling through exogenous RA supplementation, CYP26 inhibition, RAR antagonism, or inhibition of RA synthesis can alter spermatogenic progression and fertility. These mechanisms provide opportunities for development of reversible non-hormonal male contraceptives, treatment of testicular dysfunction, and fertility management. Within a One Health framework, RA-based reproductive control strategies may contribute to humane dog population management, reduced zoonotic disease transmission, improved animal welfare, conservation of wildlife, and broader human–animal–environmental health benefits.

Figure 2 summarizes the integrated retinoic acid signaling network governing spermatogenesis and illustrates the progression from vitamin A metabolism and RA homeostasis to receptor-mediated gene regulation, meiotic initiation, pharmacological intervention, and translational applications. The figure also highlights the potential contribution of RA-targeted reproductive management strategies to canine population control and broader One Health objectives, thereby linking molecular reproductive biology with veterinary, public health, and environmental outcomes.

Comparative investigations across mammalian species have established retinoic acid (RA) signaling as a highly conserved regulator of spermatogenesis while revealing important species-specific differences in reproductive physiology and pathway regulation. To synthesize the available evidence and highlight its translational relevance, Table 1 summarizes the principal experimental models, RA signaling targets, major findings, and potential applications across species.

Key Translational Themes Emerging Across Species:

1. RA signaling is highly conserved among mammals and serves as a central regulator of spermatogonial differentiation and meiotic initiation.
2. STRA8 represents the most consistently identified downstream mediator of RA-induced meiotic entry.
3. CYP26 enzymes play a critical role in regulating local RA availability and timing of spermatogenic events.
4. Pharmacological manipulation of RA signaling can reversibly alter spermatogenesis, supporting development of non-hormonal contraceptive strategies.
5. The canine model occupies a unique translational position because it combines physiological relevance, veterinary importance, and potential applications in One Health-based population management.

## 8. Key Insights, Emerging Questions and Future Directions

Retinoic acid (RA) signaling has emerged as a central regulator of postnatal spermatogenesis, integrating ligand synthesis, receptor-mediated transcription, and post-transcriptional regulation to coordinate germ cell differentiation and meiotic progression. Studies in the canine testis demonstrate that *ALDH1A2*-mediated RA synthesis, *CYP26B1*-dependent catabolism, and activation of retinoic acid receptors (RARs) function within a tightly regulated network that controls expression of key meiotic regulators, including *STRA8*, *DAZL*, and *DMRT1*. The conservation of these pathways across rodents, bovines, dogs, and humans highlights the broad biological and translational relevance of RA signaling in reproductive physiology.

### 8.1. Key Insights and Integration

Current evidence supports an integrated model in which RA availability is regulated through a dynamic balance between *ALDH1A2*-driven synthesis and *CYP26B1*-mediated degradation, creating a metabolic rheostat that maintains optimal intratesticular RA concentrations. This homeostatic system enables precise temporal regulation of meiotic initiation and germ cell differentiation. Following ligand binding, RARα, RARβ, and RARγ mediate transcriptional programs that activate downstream effectors required for spermatogenic progression. Among these, *STRA8* functions as a principal initiator of meiosis, while *DAZL* and *DMRT1* integrate RA-dependent signals with broader germ cell developmental programs. In addition to transcriptional regulation, RA signaling modulates conserved microRNA (miRNA) networks, including miR-200, let-7, and miR-34 families, which fine-tune germ cell proliferation, survival, and differentiation at the post-transcriptional level [31]. Pharmacological studies further demonstrate the plasticity of the RA system, as exogenous RA, CYP26 inhibition, and RAR antagonism differentially alter transcriptional and post-transcriptional pathways involved in spermatogenesis.

### 8.2. Critical Knowledge Gaps and Unresolved Questions

Albeit considerable advances in understanding RA-mediated spermatogenesis, several important limitations remain. Most mechanistic evidence derives from rodent models, and the extent to which these pathways operate identically in dogs remains incompletely understood. Furthermore, the long-term consequences of manipulating RA signaling on Sertoli cell function, Leydig cell physiology, sperm quality, and fertility recovery have not been adequately evaluated. The interaction between RA signaling and other regulatory pathways, including androgen signaling, growth factors, and epigenetic regulators, also requires further investigation. Future studies should focus on defining species-specific regulatory mechanisms and identifying potential adverse effects associated with prolonged modulation of RA metabolism or receptor activity.

### 8.3. Emerging Questions

Despite substantial progress, several important questions remain unresolved. The temporal precision of RA signaling during postnatal testicular development requires further clarification, particularly regarding how prolonged modulation of RA synthesis or catabolism may affect long-term fertility and Sertoli or Leydig cell function. Additional investigation is also needed to define potential non-genomic roles of RARs in post-meiotic sperm, including possible effects on sperm maturation, motility, and fertilization competence. Furthermore, the complexity of RA-responsive miRNA networks and their integration with transcriptional pathways remain incompletely understood. Although core RA signaling mechanisms are highly conserved, species-specific differences in receptor localization, spermatogenic timing, and seminiferous epithelial organization suggest the presence of distinct regulatory adaptations that warrant further comparative study.

### 8.4. Future Directions

Future research should focus on integrating advanced molecular and functional approaches to better define RA-mediated regulation of spermatogenesis. Single-cell transcriptomics, spatial profiling, and organotypic culture systems may improve understanding of stage-specific RA dynamics within the seminiferous epithelium [5,81,82,83]. Development of isoform-selective ALDH, CYP26, and RAR modulators could provide more precise tools for experimental and therapeutic manipulation of spermatogenesis. Expanding comparative analyses across canine, rodent, bovine, and human systems will further clarify conserved versus species-specific regulatory mechanisms and strengthen translational applications. Collectively, these approaches may facilitate the development of targeted therapies for testicular dysfunction and reversible non-hormonal contraceptive strategies based on modulation of RA signaling pathways.

Several emerging technologies offer exciting opportunities to advance understanding of RA signaling in spermatogenesis. Single-cell RNA sequencing may identify cell-specific responses to RA within the seminiferous epithelium, while spatial transcriptomics could define local RA gradients and receptor activity within the testicular microenvironment [84,85]. Development of canine testicular organoids may facilitate mechanistic investigations and drug screening under physiologically relevant conditions. In addition, genome-editing approaches and functional genomics could help identify novel regulators of RA signaling and reveal species-specific adaptations in reproductive biology. These approaches may accelerate development of precision reproductive therapies and next-generation contraceptive strategies.

### 8.5. Emerging Opportunities in Reproductive Biotechnology

Beyond contraception, RA signaling may have broader applications in reproductive medicine. Modulation of RA pathways could potentially improve spermatogenic recovery following gonadotoxic treatments, enhance germ-cell differentiation in vitro, and support development of regenerative approaches for male infertility. In assisted reproductive technologies, RA-responsive biomarkers may provide novel indicators of testicular function and sperm developmental competence. Furthermore, selective targeting of RA metabolic enzymes or receptor isoforms could enable individualized reproductive interventions with greater efficacy and fewer adverse effects. These emerging opportunities position RA signaling as a promising target for both fertility suppression and fertility restoration.

## 9. Dog Population Control Within the One Health Framework

The One Health framework emphasizes the interconnectedness of human, animal, and environmental health and provides a critical lens through which the global challenge of free-roaming and uncontrolled dog populations can be addressed. Dogs occupy a unique ecological and societal position as companion animals, working animals, and free-ranging community animals, resulting in complex interactions that influence disease dynamics, biodiversity, and animal welfare [86]. Rapid urbanization, insufficient veterinary infrastructure, uncontrolled breeding, abandonment, and limited public awareness have contributed to the sustained growth of stray and semi-owned dog populations in both low- and middle-income countries and, to a lesser extent, developed regions [87]. Within this context, dog population management becomes not only a veterinary concern but a multifaceted public health, ecological, and socio-political issue requiring coordinated interdisciplinary action.

From a One Health perspective, uncontrolled dog populations are strongly associated with zoonotic disease transmission, including rabies, leptospirosis, echinococcosis, and a range of parasitic infections that affect humans, livestock, and wildlife [88]. Rabies remains the most significant global concern, causing tens of thousands of human deaths annually, with free-roaming dogs acting as the principal reservoir in many endemic regions [89]. Beyond infectious disease risks, overpopulation contributes to dog bites, road traffic accidents, environmental contamination, and predation pressure on native wildlife species [90]. At the same time, free-roaming dogs often experience severe welfare challenges, including malnutrition, injury, exposure, and inhumane control practices, underscoring the ethical dimension of population management strategies [91].

Historically, population control relied heavily on lethal measures such as poisoning and mass culling. However, evidence indicates that these approaches are largely ineffective in the long term due to ecological compensation, including immigration into vacated niches, continued abandonment, and high reproductive turnover [92]. Moreover, such methods raise significant ethical concerns and can undermine public trust in veterinary and governmental institutions [87]. Contemporary One Health strategies therefore prioritize humane, sustainable, and science-based interventions that address both reproductive capacity and disease transmission.

Surgical sterilization, particularly ovariohysterectomy and castration, remains a cornerstone of dog population control [93]. When combined with mass rabies vaccination, sterilization programs reduce reproductive output, improve animal health, and mitigate behaviors associated with mating and aggression [94]. Catch–neuter–vaccinate–release (CNVR) programs have become widely adopted as humane alternatives to culling, particularly in urban environments where complete removal of free-roaming dogs is neither feasible nor socially acceptable [95]. These programs aim to stabilize populations while simultaneously reducing rabies transmission through herd immunity. However, their effectiveness depends on sustained coverage, long-term funding, and rigorous population monitoring. Modeling studies further suggest that sterilization alone may be insufficient when coverage is low or when migration and abandonment remain uncontrolled, highlighting the need for integrated, multi-component strategies [96].

Public education and responsible ownership are essential pillars of sustainable population management. Community-based initiatives promoting sterilization, vaccination, confinement, identification, and adoption can reduce abandonment and improve overall compliance with veterinary interventions [97]. Legislative frameworks, including mandatory registration, breeding regulations, and anti-abandonment laws, further strengthen control efforts when effectively enforced. Importantly, cultural perceptions of dogs vary widely, influencing the success of intervention programs; in some regions dogs are valued for guarding or utility roles, while in others they are viewed primarily as public health risks [98]. Consequently, culturally sensitive, stakeholder-driven approaches involving veterinarians, public health officials, animal welfare organizations, and local communities are essential for long-term success.

Emerging reproductive biotechnologies, including immunocontraceptive vaccines and chemical sterilants, offer promising alternatives for scalable population control, particularly in resource-limited settings. However, challenges related to efficacy, safety, duration of action, and public acceptance remain significant barriers to widespread implementation. From an ecological perspective, free-roaming dogs also exert substantial pressure on biodiversity through predation, competition, and disease transmission to wildlife, with documented impacts on threatened species in vulnerable ecosystems [99]. Interactions with wildlife further facilitate spillover of pathogens such as canine distemper virus and parvovirus, posing additional conservation risks [100,101].

Economically, uncontrolled dog populations impose considerable burdens on healthcare systems, municipal services, livestock production, and tourism, particularly through costs associated with rabies post-exposure prophylaxis and bite management [89]. Preventive One Health strategies, especially combined vaccination and reproductive control programs, are increasingly recognized as more cost-effective than reactive approaches such as culling or post-infection treatment. International agencies, including the World Health Organization, the World Organization for Animal Health, and the Food and Agriculture Organization, advocate integrated dog population management frameworks combining veterinary services, surveillance, policy development, and community engagement [102].

### RA-Based Fertility Control as a One Health Strategy

The significance of retinoic acid (RA) signaling extends beyond reproductive biology and has important implications within the One Health framework. Recent advances in non-hormonal male contraceptive development and pharmacological modulation of RA signaling have provided proof-of-concept that selective suppression of spermatogenesis can be achieved without directly disrupting systemic endocrine function, highlighting the potential of RA-targeted pathways as a novel approach to fertility control [66,103,104,105,106,107]. In the context of canine population management, the development of safe, reversible, non-surgical male contraceptives could offer an attractive alternative to permanent surgical sterilization and reduce reliance on euthanasia-based population control programs. Such approaches may be particularly valuable in regions where large free-roaming dog populations contribute to public health challenges, animal welfare concerns, and environmental impacts.

Effective fertility control has the potential to generate benefits extending far beyond reproductive management. By reducing the number of free-roaming and uncontrolled dogs, RA-based contraceptive strategies could contribute to lower transmission rates of zoonotic diseases such as rabies, decrease wildlife predation and ecosystem disturbance, reduce environmental contamination associated with unmanaged animal populations, and improve overall animal welfare through more humane population management practices [87,89,90]. Furthermore, integration of fertility control with vaccination campaigns and community-based dog management programs aligns closely with international One Health initiatives aimed at simultaneously improving animal health, protecting human populations, and preserving environmental sustainability [108,109]. Consequently, advances in RA-mediated reproductive control represent not only a promising avenue for veterinary contraception but also a potentially valuable tool for addressing interconnected challenges at the human–animal–environment interface.

Ultimately, effective dog population management requires sustained political commitment, interdisciplinary collaboration, community engagement, and locally adapted, evidence-based strategies that balance public health protection, animal welfare, ecological integrity, and societal values. Within this broader framework, RA-based fertility control may emerge as an innovative component of integrated One Health programs, linking advances in reproductive biology with practical solutions to global challenges in animal population management and zoonotic disease prevention.

## 10. Conclusions

Retinoic acid signaling represents a central regulatory framework governing spermatogenesis through tightly controlled coordination of ligand metabolism, receptor activation, and downstream gene regulation. In the canine testis, conserved mechanisms involving ALDH1A2-mediated synthesis, CYP26B1-dependent catabolism, and RAR-driven transcription converge to regulate STRA8-dependent meiotic initiation and germ cell differentiation. These processes are further refined by post-transcriptional regulation through microRNA networks, highlighting the multilayered nature of RA control. Comparative evidence across rodents, dogs, bovines, and humans underscores the evolutionary conservation of this pathway while revealing species-specific differences in timing and regulatory dynamics. Importantly, pharmacological targeting of RA signaling offers a reversible and non-hormonal strategy for male contraception, particularly relevant for canine population control within a One Health framework. Future research should focus on spatiotemporal mapping of RA gradients, receptor-specific functions, and long-term safety of pathway modulation. Collectively, RA signaling provides both mechanistic insight and translational opportunities for reproductive biology and fertility management.

## Figures and Tables

**Table 1:** Comparative evidence for retinoic acid (RA) signaling in male reproductive biology across species and its translational relevance.

Species	Experimental Model	Major RA Targets/Pathways	Principal Findings	Translational Relevance
Mouse (*Mus musculus*)	Genetic knockout models, transgenic models, pharmacological inhibition	ALDH1A1–3, CYP26B1, RARα, RARγ, STRA8, DAZL, DMRT1	Established the central role of RA in spermatogonial differentiation and meiotic initiation; STRA8 identified as a key mediator of meiotic entry; CYP26B1 regulates temporal RA availability	Foundational mechanistic understanding of RA-mediated spermatogenesis and identification of contraceptive targets
Rat (*Rattus norvegicus*)	Pharmacological models, reproductive toxicology studies	RARs, CYP26 enzymes, STRA8	Demonstrated reversible suppression of spermatogenesis following RA pathway inhibition; characterized reproductive effects of retinoid manipulation	Important preclinical model for development of non-hormonal male contraceptives
Human (*Homo sapiens*)	Testicular tissue studies, infertility investigations, transcriptomics	RARα, RARβ, RARγ, STRA8, DAZL, ALDH1A enzymes	Conserved expression of key RA signaling components in human testes; dysregulation associated with impaired spermatogenesis and infertility	Supports therapeutic targeting of RA signaling in male infertility and reproductive medicine
Dog (*Canis lupus familiaris*)	Testicular tissue analyses, immunolocalization studies, organotypic culture systems, pharmacological studies	ALDH1A isoforms, CYP26 enzymes, RARα, RARβ, RARγ, STRA8, DAZL, microRNA networks	Demonstrated developmental regulation of RA metabolic enzymes, receptor localization in germ cells and spermatozoa, and modulation of meiosis-associated pathways following RA manipulation	Translational model bridging rodent and human reproductive biology; potential platform for development of reversible fertility-control strategies and canine population management
Bull (*Bos taurus*)	Testicular developmental studies, transcriptomic analyses	ALDH1A enzymes, CYP26B1, RARs	Conserved RA-dependent regulation of germ-cell development and spermatogenic progression	Relevant to livestock fertility management and comparative reproductive biology
Pig (*Sus scrofa*)	Developmental and reproductive physiology studies	RARs, STRA8, RA metabolic enzymes	Similar RA-mediated regulation of germ-cell differentiation and testicular maturation	Useful large-animal model with physiological similarities to human reproductive biology
Non-human Primates	Experimental reproductive studies	RAR signaling pathways, STRA8, CYP26 enzymes	Confirmed conservation of RA-regulated spermatogenesis in higher mammals	Provides translational evidence supporting clinical development of RA-targeted fertility interventions

## Data Availability

Data sharing not applicable because no new data were generated.

## References

[1] Maroto M., Torvisco S.N., García-Merino C., Fernández-González R., Pericuesta E. (2025). Mechanisms of Hormonal, Genetic, and Temperature Regulation of Germ Cell Proliferation, Differentiation, and Death During Spermatogenesis. Biomolecules.

[2] Hess R.A., França L.R., Cheng C.Y. (2007). Spermatogenesis and Cycle of the Seminiferous Epithelium. Molecular Mechanisms in Spermatogenesis.

[3] de Souza A.F., Pieri N.C.G., Martins D.D.S. (2021). Step by Step about Germ Cells Development in Canine. Animals.

[4] Anjum S., Khurshid Y., Du Plessis S.S., Omolaoye T.S. (2025). Unmasking the Epigenome: Insights into Testicular Cell Dynamics and Reproductive Function. Int. J. Mol. Sci..

[5] Chakravorty A., Simons B.D., Yoshida S., Cai L. (2024). Spatial Transcriptomics Reveals the Temporal Architecture of the Seminiferous Epithelial Cycle and Precise Sertoli-Germ Synchronization. bioRxiv.

[6] Soares J.M., Avelar G.F., França L.R. (2009). The Seminiferous Epithelium Cycle and Its Duration in Different Breeds of Dog (*Canis familiaris*). J. Anat..

[7] Romagnoli S. Two Common Causes of Infertility in the Male Dog. Proceedings of the 2006 World Congress WSAVA/FECAVA/CSAVA.

[8] Morawietz J., Körber H., Packeiser E.M., Beineke A., Goericke-Pesch S. (2023). Insights into Canine Infertility: Apoptosis in Chronic Asymptomatic Orchitis. Int. J. Mol. Sci..

[9] Mogielnicka-Brzozowska M., Cichowska A.W. (2024). Molecular Biomarkers of Canine Reproductive Functions. Curr. Issues Mol. Biol..

[10] Bettegowda A., Wilkinson M.F. (2010). Transcription and Post-Transcriptional Regulation of Spermatogenesis. Phil. Trans. R. Soc. B.

[11] Álvarez-Rodríguez M., Catalán J. (2025). Molecular Mechanisms Involved in Sperm Development, Maturation, and Fertilization. Int. J. Mol. Sci..

[12] Wang J.M., Li Z.F., Yang W.X. (2022). What Does Androgen Receptor Signaling Pathway in Sertoli Cells during Normal Spermatogenesis Tell Us?. Front. Endocrinol..

[13] Helsel A., Griswold M.D. (2019). Retinoic Acid Signaling and the Cycle of the Seminiferous Epithelium. Curr. Opin. Endocr. Metab. Res..

[14] Gewiss R.L., Schleif M.C., Griswold M.D. (2021). The Role of Retinoic Acid in the Commitment to Meiosis. Asian J. Androl..

[15] Kasimanickam V.R., Kasimanickam R.K. (2013). Retinoic Acid Signaling Biomarkers after Treatment with Retinoic Acid and Retinoic Acid Receptor Alpha Antagonist (Ro 41-5253) in Canine Testis: An In Vitro Organ Culture Study. Theriogenology.

[16] Petkovich M., Chambon P. (2022). Retinoic Acid Receptors at 35 Years. J. Mol. Endocrinol..

[17] Huang P., Chandra V., Rastinejad F. (2014). Retinoic Acid Actions through Mammalian Nuclear Receptors. Chem. Rev..

[18] Berenguer M., Duester G. (2022). Retinoic Acid, RARs and Early Development. J. Mol. Endocrinol..

[19] Ma H.T., Niu C.M., Xia J., Shen X.Y., Xia M.M., Hu Y.Q., Zheng Y. (2018). Stimulated by Retinoic Acid Gene 8 (Stra8) Plays Important Roles in Many Stages of Spermatogenesis. Asian J. Androl..

[20] Anderson E.L., Baltus A.E., Roepers-Gajadien H.L., Hassold T.J., de Rooij D.G., Page D.C. (2008). Stra8 and Its Inducer, Retinoic Acid, Regulate Meiotic Initiation in Both Spermatogenesis and Oogenesis in Mice. Proc. Natl. Acad. Sci. USA.

[21] Endo T., Romer K.A., Anderson E.L., Baltus A.E., de Rooij D.G., Page D.C. (2015). Periodic Retinoic Acid-STRA8 Signaling Intersects with Periodic Germ-Cell Competencies to Regulate Spermatogenesis. Proc. Natl. Acad. Sci. USA.

[22] Kasimanickam V.R. (2016). Expression of Retinoic Acid-Metabolizing Enzymes, ALDH1A1, ALDH1A2, ALDH1A3, CYP26A1, CYP26B1 and CYP26C1 in Canine Testis during Post-Natal Development. Reprod. Domest. Anim..

[23] Maynard L.H., Humbert O., Peterson C.W., Kiem H.-P. (2021). Genome Editing in Large Animal Models. Mol. Ther..

[24] Brito M.M., da Rosa Filho R.R., Losano J.D.A., Vannucchi C.I. (2021). Ageing Changes Testes and Epididymis Blood Flow Without Altering Biometry and Echodensity in Dogs. Anim. Reprod. Sci..

[25] Nagashima J.B., Songsasen N. (2021). Canid Reproductive Biology: Norm and Unique Aspects in Strategies and Mechanisms. Animals.

[26] Slater M.R. (2001). The Role of Veterinary Epidemiology in the Study of Free-Roaming Dogs and Cats. Prev. Vet. Med..

[27] Smith L.M., Hartmann S., Munteanu A.M., Dalla Villa P., Quinnell R.J., Collins L.M. (2019). The Effectiveness of Dog Population Management: A Systematic Review. Animals.

[28] Kasimanickam V.R., Kasimanickam R.K. (2023). In Silico Analysis of miRNA-Mediated Genes in the Regulation of Dog Testes Development from Immature to Adult Form. Animals.

[29] Stukenborg J.-B. (2025). The Use of Testicular Organoids in Advancing Future Treatments for Male Factor Infertility. Fertil. Steril..

[30] Chen J., Cai T., Zheng C., Lin X., Wang G., Liao S., Wang X., Gan H., Zhang D., Hu X. (2017). MicroRNA-202 Maintains Spermatogonial Stem Cells by Inhibiting Cell Cycle Regulators and RNA Binding Proteins. Nucleic Acids Res..

[31] Kasimanickam V.R., Kasimanickam R.K., Dernell W.S. (2014). Dysregulated MicroRNA Clusters in Response to Retinoic Acid and CYP26B1 Inhibitor Induced Testicular Function in Dogs. PLoS ONE.

[32] Busada J.T., Geyer C.B. (2016). The Role of Retinoic Acid (RA) in Spermatogonial Differentiation. Biol. Reprod..

[33] Velte E.K., Niedenberger B.A., Serra N.D., Singh A., Roa-DeLaCruz L., Hermann B.P., Geyer C.B. (2019). Differential RA responsiveness directs formation of functionally distinct spermatogonial populations at the initiation of spermatogenesis in the mouse. Development.

[34] Chung S.S., Wolgemuth D.J. (2004). Role of retinoid signaling in the regulation of spermatogenesis. Cytogenet. Genome Res..

[35] Zhao D., McCaffery P., Ivins K.J., Neve R.L., Hogan P., Chin W.W., Dräger U.C. (1996). Molecular identification of a major retinoic-acid-synthesizing enzyme, a retinaldehyde-specific dehydrogenase. Eur. J. Biochem..

[36] Vernet N., Dennefeld C., Rochette-Egly C., Oulad-Abdelghani M., Chambon P., Ghyselinck N.B., Mark M. (2006). Retinoic acid metabolism and signaling pathways in the adult and developing mouse testis. Endocrinology.

[37] Amory J.K., Arnold S., Lardone M.C., Piottante A., Ebensperger M., Isoherranen N., Muller C.H., Walsh T., Castro A. (2014). Levels of the retinoic acid synthesizing enzyme aldehyde dehydrogenase-1A2 are lower in testicular tissue from men with infertility. Fertil. Steril..

[38] Elizondo G., Corchero J., Sterneck E., Gonzalez F.J. (2000). Feedback inhibition of the retinaldehyde dehydrogenase gene ALDH1 by retinoic acid through retinoic acid receptor alpha and CCAAT/enhancer-binding protein beta. J. Biol. Chem..

[39] Griswold M.D. (2016). Spermatogenesis: The commitment to meiosis. Physiol. Rev..

[40] Gewiss R., Topping T., Griswold M.D. (2020). Cycles, waves, and pulses: Retinoic acid and the organization of spermatogenesis. Andrology.

[41] Parekh P.A., Garcia T.X., Waheeb R., Jain V., Gandhi P., Meistrich M.L., Shetty G., Hofmann M.C. (2019). Undifferentiated spermatogonia regulate Cyp26b1 expression through NOTCH signaling and drive germ cell differentiation. FASEB J..

[42] Koubova J., Menke D.B., Zhou Q., Capel B., Griswold M.D., Page D.C. (2006). Retinoic acid regulates sex-specific timing of meiotic initiation in mice. Proc. Natl. Acad. Sci. USA.

[43] MacLean G., Li H., Metzger D., Chambon P., Petkovich M. (2007). Apoptotic extinction of germ cells in testes of Cyp26b1 knockout mice. Endocrinology.

[44] Kim K.H., Griswold M.D. (1990). The regulation of retinoic acid receptor mRNA levels during spermatogenesis. Mol. Endocrinol..

[45] le Maire A., Teyssier C., Balaguer P., Bourguet W., Germain P. (2019). Regulation of RXR-RAR heterodimers by RXR- and RAR-specific ligands and their combinations. Cells.

[46] Havel S.L., Griswold M.D. (2024). Temporal maturation of Sertoli cells during the establishment of the cycle of the seminiferous epithelium. Biol. Reprod..

[47] Kasimanickam V.R., Kasimanickam R.K., Rogers H.A. (2013). Immunolocalization of retinoic acid receptor-alpha, -beta, and -gamma in bovine and canine sperm. Theriogenology.

[48] Kasimanickam V., Kasimanickam R. (2014). Exogenous retinoic acid and cytochrome P450 26B1 inhibitor modulate meiosis-associated genes expression in canine testis, an in vitro model. Reprod. Domest. Anim..

[49] Bowles J., Koopman P. (2007). Retinoic acid, meiosis and germ cell fate in mammals. Development.

[50] Ocaya P.A., Elmabsout A.A., Olofsson P.S., Törmä H., Gidlöf A.C., Sirsjö A. (2011). CYP26B1 plays a major role in the regulation of all-trans-retinoic acid metabolism and signaling in human aortic smooth muscle cells. J. Vasc. Res..

[51] Laue K., Jänicke M., Plaster N., Sonntag C., Hammerschmidt M. (2008). Restriction of retinoic acid activity by Cyp26b1 is required for proper timing and patterning of osteogenesis during zebrafish development. Development.

[52] Zhou Q., Li Y., Nie R., Friel P.J., Mitchell D., Evanoff R.M., Pouchnik D.J., Banasik B.E., McCarrey J.R., Small C.L. (2008). Expression of stimulated by retinoic acid gene 8 (Stra8) and maturation of murine gonocytes and spermatogonia induced by retinoic acid in vitro. Biol. Reprod..

[53] Minkina A., Matson C.K., Lindeman R.E., Ghyselinck N.B., Bardwell V.J., Zarkower D. (2014). DMRT1 protects male gonadal cells from retinoid-dependent sexual transdifferentiation. Dev. Cell.

[54] Stoppie P., Borgers M., Borghgraef P., Dillen L., Goossens J., Sanz G., Szel H., Van Hove C., Van Nyen G., Nobels G. (2000). R115866 inhibits all-trans-retinoic acid metabolism and exerts retinoidal effects in rodents. J. Pharmacol. Exp. Ther..

[55] Kasimanickam V.R., Kasimanickam R.K. (2013). Expression of CYP26B1 and related retinoic acid signalling molecules in young, peripubertal and adult dog testis. Reprod. Domest. Anim..

[56] Bellutti L., Abby E., Tourpin S., Messiaen S., Moison D., Trautmann E., Guerquin M.J., Rouiller-Fabre V., Habert R., Livera G. (2019). Divergent roles of CYP26B1 and endogenous retinoic acid in mouse fetal gonads. Biomolecules.

[57] Schubert M., Germain P. (2023). Retinoic acid and retinoid X receptors. Cells.

[58] Hasegawa K., Saga Y. (2012). Retinoic acid signaling in Sertoli cells regulates organization of the blood–testis barrier through cyclical changes in gene expression. Development.

[59] Chung S.S., Wang X., Wolgemuth D.J. (2009). Expression of retinoic acid receptor alpha in the germline is essential for proper cellular association and spermiogenesis during spermatogenesis. Development.

[60] Hong S.-H., Castro G., Wang D., Nofsinger R., Kane M., Folias A., Atkins A.R., Yu R.T., Napoli J.L., Sassone-Corsi P. (2024). Targeting nuclear receptor corepressors for reversible male contraception. Proc. Natl. Acad. Sci. USA.

[61] Noman M.A.A., Kyzer J.L., Chung S.S.W., Wolgemuth D.J., Georg G.I. (2020). Retinoic acid receptor antagonists for male contraception: Current status. Biol. Reprod..

[62] Zhao Y., Deng S., Li C., Cao J., Wu A., Chen M., Ma X., Wu S., Lian Z. (2024). The role of retinoic acid in spermatogenesis and its application in male reproduction. Cells.

[63] Chung S.S., Wang X., Wolgemuth D.J. (2016). Prolonged oral administration of a pan-retinoic acid receptor antagonist inhibits spermatogenesis in mice with a rapid recovery and changes in the expression of influx and efflux transporters. Endocrinology.

[64] Mehrvar S., Kambara T. (2022). Morphologic features and deep learning-based analysis of canine spermatogenic stages. Toxicol. Pathol..

[65] Chien S.T., Suydam I.T., Woodrow K.A. (2023). Prodrug approaches for the development of long-acting drug delivery systems. Adv. Drug Deliv. Rev..

[66] Shi R., Wolgemuth D.J., Georg G.I. (2025). Development of the retinoic acid receptor alpha-specific antagonist YCT-529 for male contraception: A brief review. Contraception.

[67] Freeman W., Russo J. (2026). Rebalancing reproductive responsibility: Progress in nonhormonal male contraception. Pharm. Times.

[68] Zhou Y., Wang Y. (2022). Action and interaction between retinoic acid signaling and blood-testis barrier function in the spermatogenesis cycle. Cells.

[69] Fonseca P.A.S., Dos Santos F.C., Lam S., Suárez-Vega A., Miglior F., Schenkel F.S., Diniz L.A.F., Id-Lahoucine S., Carvalho M.R.S., Cánovas A. (2018). Genetic mechanisms underlying spermatic and testicular traits within and among cattle breeds: Systematic review and prioritization of GWAS results. J. Anim. Sci..

[70] Cao M., Guo S., Ding Z., Hu L., Xiong L., Ge Q., Pei J., Guo X. (2025). Comparative analysis of testicular transcriptional and translational landscapes in yak and cattle-yak: Implications for hybrid male sterility. Biomolecules.

[71] Turner L.M., Chuong E.B., Hoekstra H.E. (2008). Comparative analysis of testis protein evolution in rodents. Genetics.

[72] Yang T.A., Yang Y.H., Peng Y.H., Cong B., Diao Y.F., Bao K., Hu P.F., Song X.C., Liu L.L., Yang Y.F. (2016). Comparative studies on testicular and epididymal morphology, and serum hormone concentrations in foxes and the hybrids during the breeding season. Anim. Reprod. Sci..

[73] Griswold M.D., Hogarth C.A., Bowles J., Koopman P. (2012). Initiating meiosis: The case for retinoic acid. Biol. Reprod..

[74] Li H., MacLean G., Cameron D., Clagett-Dame M., Petkovich M. (2009). Cyp26b1 expression in murine Sertoli cells is required to maintain male germ cells in an undifferentiated state during embryogenesis. PLoS ONE.

[75] Lee W.Y., Park H.J., Lee R., Lee J.H., Jhun H., Hur T.Y., Song H. (2017). Analysis of putative biomarkers of undifferentiated spermatogonia in dog testis. Anim. Reprod. Sci..

[76] Hickford D.E., Wong S.F.L., Frankenberg S.R., Shaw G., Yu H., Chew K.Y., Renfree M.B. (2017). Expression of STRA8 is conserved in therian mammals but expression of CYP26B1 differs between marsupials and mice. Biol. Reprod..

[77] Zhang H.Z., Hao S.L., Yang W.X. (2022). How does retinoic acid (RA) signaling pathway regulate spermatogenesis?. Histol. Histopathol..

[78] Li X., Long X.-Y., Xie Y.-J., Zeng X., Chen X., Mo Z.-C. (2019). The roles of retinoic acid in the differentiation of spermatogonia and spermatogenic disorders. Clin. Chim. Acta.

[79] Harkey M.A., Asano A., Zoulas M.E., Torok-Storb B., Nagashima J., Travis A. (2013). Isolation, genetic manipulation, and transplantation of canine spermatogonial stem cells: Progress toward transgenesis through the male germ-line. Reproduction.

[80] Koilpillai J.N., Nunan E., Butler L., Pinaffi F., Butcher J.T. (2024). Reversible contraception in males: An obtainable target?. Biology.

[81] Cui L., Nie X., Guo Y., Ren P., Guo Y., Wang X., Li R., Hotaling J.M., Cairns B.R., Guo J. (2025). Single-cell transcriptomic atlas of the human testis across the reproductive lifespan. Nat. Aging.

[82] Mecca R., Tang S., Jones C., Coward K. (2024). The limitations of testicular organoids: Are they truly as promising as we believe?. Reprod. Fert. Dev..

[83] Gille A.S., Lapoujade C., Wolf J.P., Fouchet P., Barraud-Lange V. (2019). Contribution of Single-Cell Transcriptomics to the Characterization of Human Spermatogonial Stem Cells: Toward an Application in Male Fertility Regenerative Medicine?. Int. J. Mol. Sci..

[84] Chen W.B., Zhang M.F., Yang F., Hua J.L. (2024). Applications of single-cell RNA sequencing in spermatogenesis and molecular evolution. Zool. Res..

[85] Xu Q., Chen H. (2025). Applications of spatial transcriptomics in studying spermatogenesis. Andrology.

[86] Destoumieux-Garzón D., Mavingui P., Boetsch G., Boissier J., Darriet F., Duboz P., Fritsch C., Giraudoux P., Le Roux F., Morand S. (2018). The One Health concept: 10 years old and a long road ahead. Front. Vet. Sci..

[87] Taylor L.H., Wallace R.M., Balaram D., Lindenmayer J.M., Eckery D.C., Mutonono-Watkiss B., Parravani E., Nel L.H. (2017). The role of dog population management in rabies elimination—A review of current approaches and future opportunities. Front. Vet. Sci..

[88] Cleaveland S., Kaare M., Knobel D., Laurenson M.K. (2006). Canine vaccination—Providing broader benefits for disease control. Vet. Microbiol..

[89] Hampson K., Coudeville L., Lembo T., Sambo M., Kieffer A., Attlan M., Barrat J., Blanton J.D., Briggs D.J., Cleaveland S. (2015). Estimating the global burden of endemic canine rabies. PLoS Negl. Trop. Dis..

[90] Morters M.K., McKinley T.J., Horton D.L., Cleaveland S., Schoeman J.P., Restif O., Whay H.R., Goddard A., Fooks A.R., Damriyasa I.M. (2014). Achieving population-level immunity to rabies in free-roaming dogs in Africa and Asia. PLoS Negl. Trop. Dis..

[91] Matter H., Daniels T., Macpherson C.N.L., Meslin F.X., Wandeler A.I. (2000). Dog ecology and population biology. Dogs, Zoonoses and Public Health.

[92] Butcher R. Humane dog population management guidelines. Proceedings of the NAVC Conference Proceedings.

[93] Fielding W.J. (2010). Dog breeding in New Providence, The Bahamas, and its potential impact on the roaming dog population II: The fate of puppies. J. Appl. Anim. Welf. Sci..

[94] Totton S.C., Wandeler A.I., Zinsstag J., Bauch C.T., Ribble C.S., Rosatte R.C., McEwen S.A. (2010). Stray dog population demographics in Jodhpur, India following a population control/rabies vaccination program. Prev. Vet. Med..

[95] Reece J.F., Chawla S.K. (2006). Control of rabies in Jaipur, India, by the sterilisation and vaccination of neighbourhood dogs. Vet. Rec..

[96] Morters M.K., McKinley T.J., Restif O., Conlan A.J.K., Cleaveland S., Hampson K., Whay H.R., Damriyasa I.M., Wood J.L.N. (2014). The demography of free-roaming dog populations and applications to disease and population control. J. Appl. Ecol..

[97] Dalla Villa P., Kahn S., Stuardo L., Iannetti L., Di Nardo A., Serpell J.A. (2010). Free-roaming dog control among OIE-member countries. Prev. Vet. Med..

[98] Jackman J., Rowan A., Salem D.J., Rowan A.N. (2007). Free-roaming dogs in developing countries: The benefits of capture, neuter, and return programs. The State of the Animals 2007.

[99] Young J.K., Olson K.A., Reading R.P., Amgalanbaatar S., Berger J. (2011). Is wildlife going to the dogs? Impacts of feral and free-roaming dogs on wildlife populations. BioScience.

[100] Fiorello C.V., Noss A.J., Deem S.L. (2006). Demography, hunting ecology, and pathogen exposure of domestic dogs in the Isoso of Bolivia. Conserv. Biol..

[101] Costanzi L., Brambilla A., Di Blasio A., Dondo A., Goria M., Masoero L., Gennero M.S., Bassano B. (2021). Beware of dogs! Domestic animals as a threat for wildlife conservation in Alpine protected areas. Eur. J. Wildl. Res..

[102] Zinsstag J., Schelling E., Bonfoh B., Fooks A.R., Kasymbekov J., Waltner-Toews D., Tanner M. (2009). Towards a “One Health” research and application tool box. Vet. Ital..

[103] Garcia T.X., Matzuk M.M. (2024). Novel Genes of the Male Reproductive System: Potential Roles in Male Reproduction and as Non-hormonal Male Contraceptive Targets. Mol. Reprod. Dev..

[104] Balbach M., Rossetti T., Ferreira J., Ghanem L., Ritagliati C., Myers R.W., Huggins D.J., Steegborn C., Miranda I.C., Meinke P.T. (2023). On-demand male contraception via acute inhibition of soluble adenylyl cyclase. Nat. Commun..

[105] Mariani N.A.P., Silva J.V., Fardilha M., Silva E.J.R. (2023). Advances in non-hormonal male contraception targeting sperm motility. Hum. Reprod. Update.

[106] Nickels L., Yan W. (2023). Nonhormonal Male Contraceptive Development-Strategies for Progress. Pharmacol. Rev..

[107] Kent K., Johnston M., Strump N., Garcia T.X. (2020). Toward Development of the Male Pill: A Decade of Potential Non-hormonal Contraceptive Targets. Front. Cell Dev. Biol..

[108] Nadal D., Bote K., Abela B. (2023). Is there hope to reach the Zero by 30 target for dog-mediated human rabies?. Lancet Glob. Health.

[109] Tidman R., Fahrion A.S., Thumbi S.M., Wallace R.M., De Balogh K., Iwar V., Yale G., Dieuzy-Labaye I. (2023). United Against Rabies Forum: The first 2 years. Front. Public Health.

